# The Endocannabinoid System in the Mediterranean Mussel *Mytilus galloprovincialis*: Possible Mediators of the Immune Activity?

**DOI:** 10.3390/ijms22094954

**Published:** 2021-05-07

**Authors:** Francesco Mosca, Osvaldo Zarivi, Natalia Battista, Mauro Maccarrone, Pietro Giorgio Tiscar

**Affiliations:** 1 Faculty of Veterinary Medicine, University of Teramo, 64100 Teramo, Italy; fmosca@unite.it (F.M.); pgtiscar@unite.it (P.G.T.); 2 Center of Integrated Research, University of L’Aquila, 67100 L’Aquila, Italy; osvaldo.zarivi@univaq.it; 3 Faculty of Bioscience and Technology for Food, Agriculture and Environment, University of Teramo, 64100 Teramo, Italy; 4 Department of Biotechnological and Applied Clinical Sciences, University of L’Aquila, 67100 L’Aquila, Italy; mauro.maccarrone@univaq.it; 5 European Center for Brain Research, IRCCS Santa Lucia Foundation, 00164 Rome, Italy

**Keywords:** anandamide, cannabinoid receptors, evolution, hemocytes, mussel, vanilloid receptors

## Abstract

Anandamide (AEA) is one of the best characterized members of the endocannabinoid family and its involvement in many pathophysiological processes has been well documented in vertebrates and invertebrates. Here, we report the biochemical and functional characterization of key elements of the endocannabinoid system in hemocytes isolated from the Mediterranean mussel *Mytilus galloprovincialis*. We also show the effects of exogenous AEA, as well as of capsaicin, on the cell ability to migrate and to activate the respiratory burst, upon in vitro stimulation of phagocytosis. Interestingly, our findings show that both AEA and capsaicin suppress the hemocyte response and that the use of selective antagonists of CB_2_ and TRPV1 receptors revert their inhibitory effects. Overall, present data support previous evidence on the presence of endocannabinoid signaling in mollusks and advance our knowledge about the evolutionary origins of this endogenous system and its role in the innate response of mollusks.

## 1. Introduction

Anandamide (AEA), together with 2-arachidonoylglycerol (2-AG), is the most biologically active member of a large family of polyunsaturated fatty acid amides, esters, and ethers collectively known as endocannabinoids (eCBs), recently included in a larger network defined “endocannabinoidome” [1]. The biological activity of eCBs is tightly controlled by metabolic enzymes that synthesize and cleave AEA (*N*-acylphosphatidylethanolamines-specific phospholipase D (NAPE-PLD), and fatty acid amide hydrolase (FAAH), respectively) [2], or 2-AG (diacylglycerol lipases (DAGL) α and β, and monoacylglycerol lipase (MAGL), respectively) [3]. Among the eCB-like compounds, *N*-palmitoylethanolamine (PEA) exerts anti-inflammatory properties and, while its synthetic metabolic route is still under investigation, its catabolism has been ascribed to a member of choloylglycine hydrolase family, called *N*-acylethanolamine hydrolyzing amide amidase (NAAA) [4]. The eCBs act by binding to type-1 and type-2 cannabinoid receptors (CB_1_ and CB_2_), both members of the rhodopsin-like GPCR family, to the G protein coupled orphan receptor 55 (GPR55) and to transient receptor potential vanilloid type 1 (TRPV1) [5]. Together with their metabolic enzymes, membrane transporters (EMT) and molecular targets, eCBs form the endocannabinoid system (ECS) [5]. Although AEA was identified for the first time in 1992, it seems to be part of an ancient signaling network that has been conserved for more than 500 million years [6,7,8,9]. Of note, AEA appeared much earlier than the cannabis (*Cannabis sativa*)-derived analogues like ∆^9^-tetrahydrocannabinol (THC) and other phytocannabinoids [10]. The presence of eCBs and various elements of ECS in cells and tissues of invertebrates, vertebrates, and mammals highlighted their value along the evolutionary axis [8,9,11,12], even though their physiological and functional roles at the low branches of the phylogenetic tree still remain to be understood. Binding assays with radiolabeled ligands have shown that cannabinoid receptors evolved in basal animals such as Cnidarian (*Hydra vulgaris*), Nematode (*Panagrellus redivivus*), and Porifera (*Tethya aurantium*) [13]. A putative ancestral cannabinoid receptor has been identified in the deuterostomic invertebrate *Ciona intestinalis* [14], while the biochemical pathways for the biosynthesis and inactivation of both AEA and 2-AG have been reported in the sea urchin *Strongylocentrotus purpuratus* [15] and in *Hirudo medicinalis* [16]. Despite the relevance of mollusks as a model system in neurobiology, only few biochemical studies have been focused on ECS, demonstrating specific AEA binding sites in *Mytilus edulis spp.* [17] and the presence of AEA and FAAH in the Mediterranean mussel *Mytilus galloprovincialis* [18]. More recently, in the mollusk *Lymnaea stagnali* mRNAs encoding for two CB-like GPCRs (i.e., LymCBR-like 1 and 2) have been identified suggesting the existence of putative cannabinoid receptors in protostomic invertebrates [19]. Some studies suggest that, although there is limited sequence similarity, some of these putative CBRs show substantial pharmacological overlap with CB_1_ and CB_2_ [20]. Although CB_1_/CB_2_-type receptors are unique to chordates, enzymes involved in the biosynthesis / inactivation of eCBs are present throughout the animal kingdom. Many components of the ECS function as key regulators of the immune system and the immune response [21,22]; indeed, it has been demonstrated that eCBs can stimulate migration of human hematopoietic stem cells through a cannabinoid receptor-dependent manner, are involved in the regulation of mature immune cell trafficking and effector cell functions as well as act as chemotactic substances capable of recruiting dendritic cells during the innate immune response [21]. The role of mussel hemocytes as immunocompetent cells, particularly involved in the phagocytosis process, has been widely investigated [23,24] but, despite few evidences obtained on the control by 2-AG of the immunosuppressive response in immunocytes from *Mytilus edulis* [25], there are still few evidences on the involvement of ECS in molluscan immunity. Here, we show the presence of the main ECS components in hemocytes from the Mediterranean mussel *Mytilus galloprovincialis*, providing a novel contribution to comparative biology studies and suggesting a potential function of this signaling system in the innate immune response of bivalve molluscan species.

## 2. Results

### 2.1. Characterization of the ECS in Mussel Hemocytes

The presence of AEA, 2-AG, and PEA, along with the activity of the ECS elements involved in their metabolism, were tested by biochemical assays, demonstrating that all expected enzymes were detectable and functional in hemocytes from the Mediterranean mussel *Mytilus galloprovincialis* (Table 1).

Since the mechanism of PEA biosynthesis is not yet clear, we focused on the activity of NAAA, the putative enzyme responsible for PEA degradation, showing that the hydrolysis of [^3^H]PEA at pH 5.0 was 97 ± 20 pmol/min per mg of protein. Intact hemocytes were able to accumulate [^3^H]AEA in a concentration dependent manner, according to a saturable process typical of EMT, showing apparent Km and Vmax values of 691 ± 100 nM and 15.6 ± 1 (pmol/min per 10^6^ cells), respectively. Next, we investigated the ability of hemocytes to take up 2-AG with a saturable process, showing kinetic constants in the same range as those calculated for AEA (data not shown). Moreover, the full inhibition of AEA and 2-AG transport by OMDM1 (10 μM) showed that these eCBs are likely driven into the cells through the same carrier.

### 2.2. Cannabinoid and Vanilloid Receptors in Mussel Hemocytes

**Figure 1:**
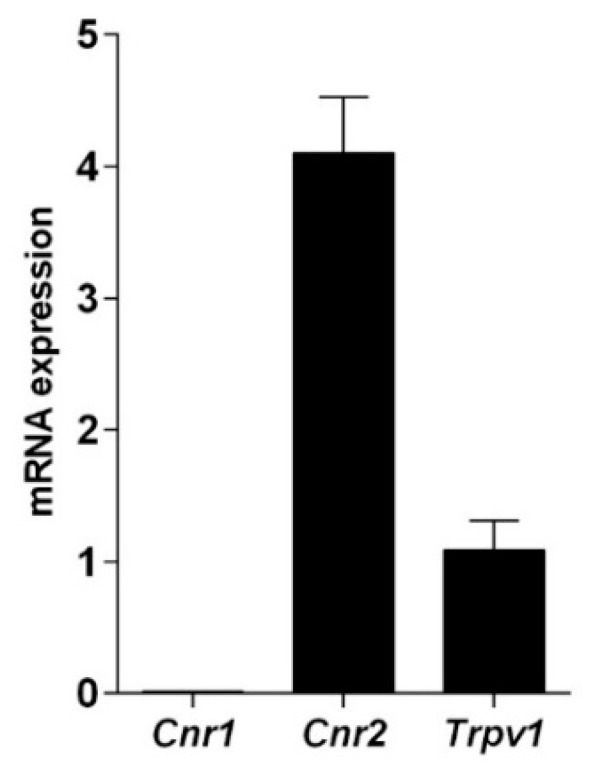
Quantitative RT-PCR analysis of the mRNA levels of cannabinoid and vanilloid receptors. The mRNA levels are expressed as fold over *Trpv1* set to 1. Gene expression data are reported as 2^−ΔΔCt^ values calculated by Delta-Delta Ct method versus *Trpv1* posed equal to 1.

The presence of cannabinoid and vanilloid receptor was tested at transcriptional level and their functionality was verified through binding assays performed with the synthetic CB_1_ and CB_2_ agonist [^3^H]CP55.940. qRT-PCR data (Figure 1) showed that these cells express CB_2_ and TRPV1, while CB_1_ is barely detectable, as calculated by the threshold cycle (C_t_) values (*Cnr1* = 31.54 ± 0.31; *Cnr2* = 23.34 ± 0.09; *Trpv1* = 25.39 ± 0.06; β-actin = 23.40 ± 0.25).

Incidentally, it should be underlined that specific amplification products were obtained only when human, but not mouse and rat, primers were used for DNA recognition. Consistently with biochemical data, functional data demonstrated that the synthetic cannabinoid [^3^H]CP55.940 was bound dose-dependently to hemocytes, showing an saturation curve overlapping to that obtained with mouse spleen membranes, a positive control for CB_2_. The values of the binding kinetic constants K_d_ = 356 ± 160 pM and B_max_ = 16 ± 3 fmol/mg of protein confirmed the presence of CB_2_ receptors in these immune cells. In addition, the binding of the synthetic cannabinoid [^3^H]CP55.940 was displaced down to ~70% by SR144528, a selective CB_2_ antagonist, but not by 0.1 μM SR141716, a selective CB_1_ antagonist. Moreover, hemocytes were able to bind [^3^H]RTX according to a saturable process, showing apparent K_d_ of 413 ± 168 pM and B_max_ of 77 ± 11 fmol/mg, suggesting thus that hemocytes possess also vanilloid receptors. 

### 2.3. Phylogenetic Study of ECS

**Figure 2:**
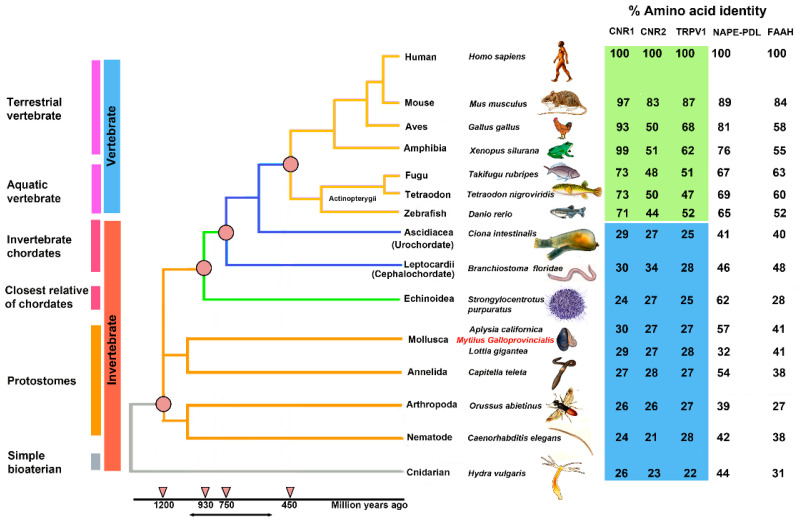
A phylogenetic “Tree of Life” of ECS in 15 species of vertebrates (blue) and invertebrates (orange), divided into six physiological groups: terrestrial vertebrate, aquatic vertebrate, invertebrate chordates, closest relative of chordates, protostomes, and simple bilaterian.

Figure 2 shows a phylogenetic “Tree of Life” with the evolutionary relationships of 15 species and the percentage of identity for each amino acid sequence identified in the analysis (Supplementary Table S1).

**Figure 3:**
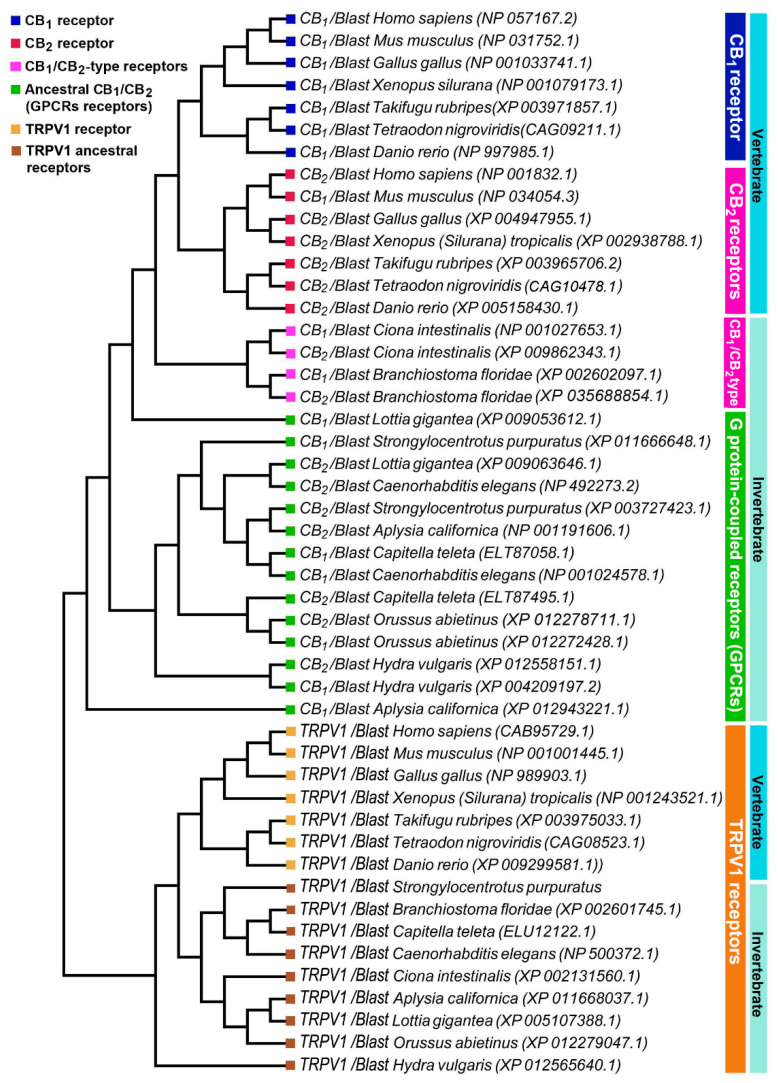
Phylogenetic analysis of vertebrate and invertebrate cannabinoid CB_1_, CB_2_, and TRPV1 receptors.

The percentages of identity of the identified sequences highlights that CB_1_ and CB_2_ receptors began their evolution at the level of the Bilatera branch from a common ancestral receptor about one thousand million years ago, assuming their definitive function about five hundred million years ago in the Deuterostomes (aquatic vertebrates). Interestingly, the percentage of identity for cannabinoid receptors in the transition from vertebrates to invertebrates shows a marked difference: *Danio rerio* shares approximately 71% sequence identity for CB1 compared to *Ciona intestinalis* 29%, whereas the values are lower for CB_2_: 44% in *Danio rerio* versus 27% in *Ciona intestinalis*. This difference is consistent with the literature data reporting that the origin of the receptors is a possible consequence of a gene duplication of a common ancestor (GPCRs) already expressed in invertebrates. The phylogenetic tree of Figure 3 shows distinct groups in vertebrates for CB_1_ (blue square) and CB_2_ (red square).

**Figure 4:**
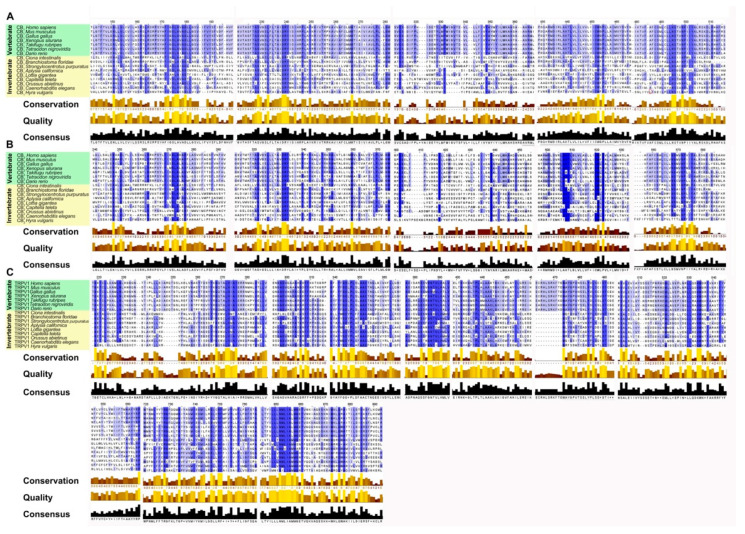
Alignment of multiple sequences of the functional orthologs of cannabinoid receptors (CB_1_, CB_2_, TRPV1) of 15 different species generated by Tcoffell. The refinements were carried out with the Jalview software. For better comparability, all sequences were shortened to emphasize the conserved domains. The highlighted boxes represent a preserved pattern and the intensity of the blue color is related to the degree of conservation. In addition, the conservation, quality (yellow to brown bars) and consensus (black bars) sequences are shown. (**A**) Alignment of functional orthologs of the CB_1_ receptor. (**B**) Alignment of functional orthologs of the CB_2_ receptor. (**C**) Alignment of functional orthologs of the TRPV1 receptor.

To highlight the evolutionary transition point from an ancestral GPCR receptor to the CB receptors, we gathered the CB_1_/CB_2_-type receptors of *Ciona intestinalis* and *Branchiostoma floridae* in a single clade (pink square). Interestingly, in invertebrates there is a single clade where all the sequences obtained from Blast analysis converge, this makes us hypothesize the presence of a single ancestral GPCRs receptor, which gave rise to CB_1_ and CB_2_. TRPV1 receptors form a distinct group from CB receptors with a single clade for vertebrates and invertebrates, demonstrating that TRPV1 is a different receptor than CB_1_ and CB_2_ (Figure 3). The diversity of motifs of the amino acids conserved in TRPV1 compared to CB_1_/CB_2_ was also supported by the analysis with the Jalview software (Figure 4).

BLAST analysis for the enzymes involved in the biosynthesis (NAPE-PLD) and inactivation (FAAH) of AEA highlighted a gradual evolution over time with decreasing percentages of identities along the time span of the phylogenetic tree (Figure 2).

### 2.4. Effect of AEA on Phagocytosis Assay

**Figure 5:**
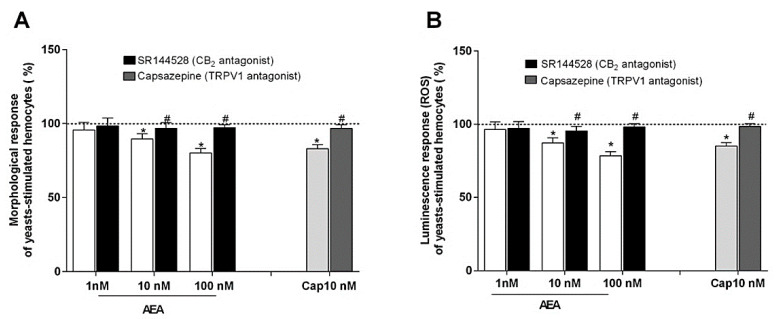
Effects of AEA and CAP on the hemocyte ability (**A**) to undergo morphological activation and (**B**) to generate ROS following phagocytosis stimulation with yeasts. AEA and CAP were tested both alone and in combination, respectively, with SR144528, a CB_2_ antagonist, and CPZ, a TRPV1 antagonist. Control (dotted line) was represented by yeasts-stimulated hemocytes not exposed to AEA or CAP and their respective antagonists. Control = 100%; * *p* < 0.01 vs. control; ^#^ *p* < 0.01 vs. hemocyte response w/o antagonist.

In these experiments, the effects of AEA on the hemocyte ability to spread during phagocytosis stimulation were tested. Interestingly, AEA induced a dose-dependent inhibition of this activity and this decrease was partially reverted by the treatment with SR144528, the inverse CB_2_ agonist. In addition, capsaicin (CAP), the TRPV1 agonist, was also able to reduce the morphological activity of the stimulated hemocytes and the inhibitory effect was attenuated by the specific TRPV1 antagonist capsazepine (CPZ) (Figure 5A). As observed for the circularity, the incubation of the hemocytes with AEA induced a dose-dependent depression of their oxidative activity following phagocytosis stimulation, and the inhibitory effects were partially reversed by treatment with SR144528. Additionally, the incubation with CAP reduced the luminescence generation of the stimulated hemocytes and the effect was partially reverted by treatment with CPZ (Figure 5B).

## 3. Discussion

In this paper we showed, through molecular and immunochemical analyses, that hemocytes isolated from the Mediterranean mussel *Mytilus galloprovincialis* not only have the appropriate tools to synthesize, degrade, and transport the most prominent eCBs, but they also express functional eCBs-binding CB_2_ and TRPV1, but not CB_1_, receptors. In the last years a wide distribution of homologs, orthologs, and paralogs genes able to code for the ECS has been evidenced through in silico studies, that showed similarities in the genome of phylogenetically diverse organisms from bacteria to eukaryotes [8,10,26]. CB_1_ and CB_2_, paralogues in vertebrates, have undergone a divergent evolution in a short evolutionary time, which led them to share an amino acid sequence identity of about 44% [27]. Indeed, CB_1_ has a higher sequence stability than CB_2_ as demonstrated by the percentage identity in vertebrates that goes from 100% to 71% for CB_1_ and from 100% to 44% for CB_2_. On this basis, we can hypothesize that the greater sequence stability of CB_1_ is related to a more important functional role and/or to a greater affinity for the ligands. Assuming that *Ciona intestinalis* cannabinoid receptor (CiCBR) is the first cannabinoid receptor identified in an invertebrate species, we performed a similarity analysis between the CiCBR sequence and human sequences and we found an identity equal to 29% and 27%, respectively, for CB_1_ and CB_2_. Although the invertebrate sequences have low identity percentages compared to humans, the analysis performed by using Pfam and Prosite Software highlighted that the presence of the domain belongs to the G protein-coupled receptor family, rhodopsin-like in all the investigated invertebrate species, and that the domains were conserved (Figure 3), leading to assume that CiCBR is an orthologue of the vertebrate cannabinoid receptors that is evolved from the GPCR family. Analogously, TRPV1 receptor acquired its specificity in an evolutionary time window similar to that of CB_1_ and CB_2_, as shown by the values of the percentages of amino acid identity as well as from the comparison among the sequence identities CB_1_/TRPV1, CB_2_/TRPV1, and CB_1_/CB_2_ equal to 24.89%, 24.27%, and 44.50%, respectively. The study of the multiple alignment of the TRPV1 sequences, reported in Figure 4, confirms the diversity of the conserved amino acids’ motifs compared to the CB_1_ and CB_2_ sequences, suggesting that, even though the developmental age of TRPV1 and CB receptors was similar, the vanilloid receptor evolved as a different receptor from CB_1_ and CB_2_. This diversity is also confirmed by the phylogenetic tree shown in Figure 2 where TRPV1 is positioned in a distinct clade outside the CB receptors. These evidences suggest that TRPV1 receptor had an evolutionary pattern similar to CB_2_ starting from *Danio rerio* to man, including also an additional evolutionary step. These evidences confirm the hypothesis that CB_1_, CB_2_, and TRPV1 receptors appeared simultaneously in the evolutionary scale at the level of Urocordates, thus achieving full evolution in vertebrates. BLAST analysis of the enzymes involved in the AEA metabolism as reported in the phylogenetic tree, highlights an ancient and important role of the eCBs progenitor in the course of phylogeny. On this basis, we might suggest that, although CB_1_ and CB_2_ receptors are unique to chordates, the enzymes that regulate the AEA metabolism have evolved independently and are present throughout the animal kingdom, supporting the hypothesis of a non CB_1_/CB_2_ mediated endocannabinoid signaling. Based on this, we corroborate the hypothesis that both CB receptors emerged, following gene duplication and the acquisition of specific functions, from an ancestral GPCR that evolved as CiCBR and BfCBR in the last evolutionary step. Despite these data, only functional analyses might be considered effective in order to avoid false positive and our results are a straight evidence of a complete and fully functional ECS in primary hemocytes from the Mediterranean mussel *Mytilus galloprovincialis*. Although previous results reported the presence of stereoselective and monophasic binding sites with high affinity for AEA in immunocytes from *Mytilus edulis* [17], it is not surprising that CB_1_ was missing in a different species. In fact, morphological uniformity in geographically widespread species may cause genetically distinct entities that can be detected by molecular approaches and can be of critical importance for a complete understanding of ongoing and future population dynamics [28].

eCBs have been extensively examined for their immunomodulatory and anti-inflammatory effects [29,30] as well as for their involvement in the impairment of the chemotaxis activity of phagocytic cells [31,32]. The present study revealed the inhibitory effects of AEA on the hemocyte ability to spread following phagocytosis stimulation and the role played by CB_2_ in affecting cell shape. These findings are in line with previous studies showing the morphological shift, from amoeboid to rounded shape, in mussel hemocyte after AEA and 2-AG exposure [17], and reporting that eCBs might exert their biological actions via coupling to nitric oxide (NO) generation, that is crucial to maintain immune homeostasis and other physiological processes [25]. In association with the inhibition of the hemocyte morphological spreading, the phagocytosis assays carried out in the present study revealed the ability of AEA to modulate the respiratory burst with a decrease of the luminescence generation. Use of the inverse CB_2_ receptor agonist SR144528 led to partial recuperation of reactive oxygen species (ROS) production, confirming the functional role of CB_2_. Phagocytosis is the first line of internal defense in invertebrates, and phagocytosis by hemocytes is considered to be the main cellular mediated immune mechanism of shellfish. In molluscan hemocytes this process proceeds through sequential well-defined stages, including attraction or random collision, adherence (non-self recognition), endocytosis, and intracellular digestion. Although several studies showed that cannabinoids have inhibitory effects on phagocytic cells [33,34], other reports demonstrated a stimulatory effect on the phagocytosis process, probably related to the potential role of the cannabinoids on the eicosanoid synthesis [35,36]. In this context, it has been reported that nanomolar concentrations of CP55.940, but not AEA, stimulated the burst reaction of whole-blood human polymorphonuclear neutrophils (PMNs) through a CB_2_-evoked COX-dependent mediator pathway induced by cannabinoid interactions with other blood cells [37]. The apparent contradictions between stimulatory and inhibitory effects of cannabinoids on phagocytic cells have been correlated to a well-known biphasic response which can be observed by using different ligand concentrations [38]. Indeed, many of the inhibitory effects of cannabinoids in vitro have been described in the micromolar concentration range, whereas stimulatory concentrations have been observed in the nanomolar range [39]. Under our experimental conditions, the suppressive effects of AEA on hemocyte phagocytosis were observed at 10 nM and 100 nM in a dose-dependent manner, whereas the lowest concentration (1 nM) appeared as ineffective. On this basis, we suggest that the discrepancies among several studies might be ascribed to the use of different types and doses of cannabinoid ligands, experimental protocols and in vitro phagocytosis conditions. Additionally, it should be recalled that CP55.940 displays a strong signaling bias for the CB_2_, displaying a significantly greater preference for the activation of cAMP signaling better than other signal transduction pathways of the same receptor (i.e., pERK), thus, the signaling-specific preference could also be taken into account when studying the functional selectivity of the ligands towards CB_2_ receptor [40,41].

Here, we also revealed the inhibitory impact of CAP on the hemocyte phagocytosis through the activation of TRPV1, as corroborated by the data obtained following the treatment with CPZ. To our knowledge, the presence and functionality of a vanilloid system in marine bivalve mollusks has not been investigated to date, despite TRP channels having been found throughout animal kingdom including invertebrates [42]. Although the inhibitory effects of CAP on the hemocyte phagocytosis were reversed by CPZ, indicating the potential role of TRPV1 in the regulation of the hemocyte response, we cannot exclude a TRPV1-independent pathway in the modulation of innate immunity, as already described in higher vertebrates [43,44]. Moreover, we should recall the ability of CAP to induce apoptosis in peripheral blood mononuclear cells and the use of TRPV1 antagonist to be fully effective in inhibiting this effect [45]. On this basis, we can suppose that the decrease of the hemocyte phagocytosis could rely on cytotoxic activity of CAP rather than inhibition of specific inflammatory pathways, in line with the market application of this compound as a natural biocide for marine invertebrates [46]. Away from the potential activity on immune system, TRPV1 has a complex polymodal activation profile in pain signal transduction, being responsive not only to chemicals but also to thermal and mechanical stimulations [47]. Therefore, the presence of a vanilloid receptor in circulating hemocytes of marine bivalve mollusks may be intended more generally as an ancestral strategy to sense and integrate different types of information about the surrounding environment. Taken all together, the research on endocannabinoid signaling could be crucial to investigate fundamental processes associated with marine organisms, considering that marine model systems could provide new insight into basic biological principles that will benefit further development for medicine and industry.

## 4. Materials and Methods

### 4.1. Samples Collection

Mediterranean mussels *Mytilus galloprovincialis* (Lmk 1819) were kindly supplied by local producers and were maintained in the laboratory for 24 h before the hemolymph collection. The mussels were kept in a re-circulating seawater system at controlled temperature (18 °C) and salinity (33‰). Each hemolymph pool was obtained from at least 20 mussels by puncturing their posterior adductor muscle and maintained on ice to avoid hemocyte aggregation.

### 4.2. Chemicals

Anandamide (*N*-arachidonoylethanolamine, AEA), 5-(1,1-dimethylheptyl)-2-[5-hydroxy-2-(3-hydroxypropyl)cyclohexyl]phenol (CP55.940), 2-oleyl glycerol (2-OG); capsazepine (*N*-[2-(4-chlorophenyl)ethyl]-1,3,4,5-tetrahydro-7,8-dihydroxy-2H-2-benzazepine-2-carbothioamide; CPZ), were purchased from Sigma Chemical Co. (St. Louis, MO, USA). 2-Arachidonoylglycerol (2-AG) and resiniferatoxin (RTX) were purchased from Alexis Corporation (San Diego, CA, USA). *N*-Piperidino-5-(4-chlorophenyl)-1-(2,4-dichloro-phenyl)-4-methyl-3-pyrazole carboxamide (SR141716) and *N*-[(1S)-endo-1,3,3-trimethy-1-bicyclo [2.2.1]-heptan-2-yl]5-(4-choro-3-methyl-phenyl)-1-(4-methyl-benzyl)-pyrazole-3-carboxamide (SR144528) were kind gifts of Sanofi-Aventis Recherche (Montpellier, France). 5′-iodoresiniferatoxin (I-RTX) was from Tocris-Cookson (Bristol, UK). 1-Stearoyl-2-Arachidonoyl-*sn*-Glycerol was from Alexis Biochemicals (Lausen, Switzerland). AEA-ethanolamine-1-[^3^H] (60 Ci/mmol), [^3^H]CP55.940 (126 Ci/mmol) and [^3^H]RTX (43 Ci/mmol) were purchased from Perkin Elmer Life Sciences (Boston, MA, USA). 1-Stearoyl-2-Arachidonoyl[1-^14^C]-*sn*-Glycerol (55 Ci/mol), 2-oleoyl[^3^H]glycerol (20 Ci/mmol), AEA-ethanolamine-1-[^3^H] (60 Ci/mmol) and *N*-[^3^H]arachidonoyl-phosphatidylethanolamine ([^3^H]NArPE, 200 Ci/mmol) were from ARC (St. Louis, MO, USA). URB597, URB602, d_8_-AEA, d_8_-2-AG, d_4_-PEA, rabbit anti-CB_1_ and anti-CB_2_ polyclonal antibodies were from Cayman Chemicals (Ann Arbor, MI, USA). Rabbit anti-TRPV1 polyclonal antibody and goat anti-rabbit antibodies conjugated to horse radish peroxidase were purchased from Santa Cruz Biotechnology (Santa Cruz, CA, USA). Rabbit anti-β-actin polyclonal antibody was purchased from Cell Signalling Technology (Danvers, MA, USA). The selective inhibitor of AEA transport OMDM1 was a gift of Dr. Marinelli (European Center for Brain Research, Italy) and the selective inhibitor of MAGL enzyme O-3841 was kindly provided by Dr. Di Marzo (CNR Naples, Italy and Université Laval, Canada).

### 4.3. Endogenous Levels of AEA, 2-AG and PEA

Hemocytes were subjected to lipid extraction with chloroform/methanol (2:1, *v*/*v*), in the presence of d_8_-AEA, d_8_-2-AG, and d_4_-PEA, as internal standards. The organic phase was dried and then analyzed by liquid chromatography-electrospray ionization mass spectrometry (LC-ESI-MS), using a single quadrupole API-150 EX mass spectrometer (Applied Biosystem, CA, USA) in conjunction with a Perkin Elmer LC system (Perkin Elmer, MA, USA). Quantitative analysis was performed by selected ion recording over the respective sodiated molecular ions.

### 4.4. Endocannabinoid Metabolism

The synthesis of [^3^H]AEA through the activity of NAPE-PLD (E.C. 3.1.4.4) and the hydrolysis of [^3^H]AEA by FAAH (E.C. 3.5.1.4) were assayed in hemocyte homogenates, by using 100 µM [^3^H]NArPE and 10 µM [^3^H]AEA, respectively, as reported [48]. FAAH activity and its apparent Km, Vmax, and Ki values were determined in hemocytes, as reported [49]. [^3^H]AEA or [^3^H]2-AG uptake was measured on cell suspensions, as previously described [50]. Cells were incubated with the labeled compound at 4 °C and 37 °C, and the 4 °C values were subtracted from those at 37 °C. The effect of OMDM1, a selective inhibitor of the EMT, was determined by adding the compound directly to the incubation medium. DAGL activity was assayed in cell homogenate (50 µg protein/test) using 10 µM [^14^C]DAG, while MAGL was tested in cell supernatants (75 µg protein/test), using 10 µM [^3^H]2-OG as substrate. The effect of O-3841, that inhibits DAGL enzyme, and URB 602, the best and most characterized MAGL inhibitor, was ascertained under the same experimental conditions. The degradation of [^3^H]PEA by NAAA activity was assayed in tissue homogenates (100 µg/test for NAAA) by measuring the release of [^3^H]ethanolamine.

### 4.5. mRNA Expression of CB_1_, CB_2_ and TRPV1

RNA was extracted from hemocytes cells using the RNeasy extraction kit (Qiagen, Crawley, UK), as suggested by the manufacturer. Quantitative real time reverse transcriptase-polymerase chain reaction (qRT-PCR) assays were performed using the SuperScript III Platinum Two-Step qRT-PCR Kit (Invitrogen, Carlsbad, CA, USA), according to the manufacturer’s instructions. The target transcripts were amplified by means of an ABI PRISM 7700 sequence detector system (Applied Biosystems, Foster City, CA, USA), using the following primers:
**Gene****Forward (5′→3′)****Reverse (3′→5′)***Cnr1*CCTTTTGCTGCCTAAATCCACCCACTGCTCAAACATCTGAC*Cnr2*TCAACCCTGTCATCTATGCTCAGTCAGTCCCAACACTCATC*Trpv1*TCACCTACATCCTCCTGCTCAAGTTCTTCCAGTGTCTGCC*β-actin*TGACCCAGATCATGTTTGAGTTAATGTCACGCACGATTTCC

Relative mRNA expression levels were calculated using the 2^−DDCt^ method and normalized to β-Actin gene expression.

### 4.6. Receptor Binding Assays

Cannabinoid and vanilloid receptor studies were performed by rapid filtration assays, using the synthetic cannabinoid [^3^H]CP55.940 and the TRPV1 agonist [^3^H]RTX, respectively. Unspecific binding was determined in the presence of cold agonists (1 µM CP55.940 or 1 μM RTX) and was further validated by selective antagonists (0.1 μM SR141716 for CB_1_, 0.1 μM SR144528 for CB_2_ and 1 μM CPZ for TRPV1). 

### 4.7. Phylogenetic Study

The phylogenetic study included the search for enzymes involved in the biosynthesis and inactivation of AEA (NAPE-PLD and FAAH), and of functional orthologs of cannabinoid receptors (CB_1_, CB_2_, TRPV1). The evolution of the ECS was investigated in 15 phylogenetically different species representing important phylogenetic nodes: Mouse (*Mus musculus*), Aves (*Gallus gallus*), Amphibia (*Xenopus silurana*), Fugu (*Takifugu rubripes*), Tetraodon (*Tetraodon nigroviridis*), Zebrafish (*Danio rerio*), Ascidiacea (*Ciona intestinalis*), Leptocardi (*Branchiostoma floridae*), Echinoidea (*Strongylocentrotus purpuratus*), Mollusca (*Aplysia californica, Lottia gigantea*), Annelida (*Capitella teleta*), Arthropoda (*Orussus abietinus*), and Nematode (*Caenorhabditis elegans*) and Cnidaria (*Hydra vulgaris*). Searches were performed using the Basic Local Alignment Search Tool (BLAST) algorithm in the NCBI (National Center for Biotechnology Information) database, (https://blast.ncbi.nlm.nih.gov/Blast.cgi, accessed on 19 February 2021), using *Homo sapiens* query sequences. Blast analysis and multiple sequence alignments were used to identify, for each species of vertebrate and invertebrate studied, the protein sequences with the highest percentage of identity (Supplementary Table S1). For each sequence, the functional domains were studied with Software Pfam (Protein families database) and Prosite (Protein domains database), as already reported [51,52]. Multi-alignments and phylogenetic tests were performed with Clustal Omega (Multiple sequence alignment EMBL-EBI) software, Jalview Software, a program for multiple sequence alignment editing, visualization, and analysis, and MEGA6 (Molecular Evolutionary Genetics Analysis), as described previously [53,54,55].

### 4.8. Phagocytosis Assay

The phagocytosis activity of mussel hemocytes was monitored by means of in vitro assays [56] to verify the effects of AEA and CAP on the cell ability to spread and to generate ROS following stimulation with Zymosan A (Sigma-Aldrich Co., St. Louis, MO, USA). The spreading activity of the hemocytes was measured as morphological variations of the cell roundness (circularity) by means of Vi-Cell^®^ (Beckman Coulter, Milan, Italy). Each hemolymph pool was adjusted to 10^6^ hemocytes/mL with ice cold artificial seawater (ASS) and then it was divided in different aliquots. All aliquots were incubated with Zymosan A for 30 min before analysis. Some aliquots were also incubated with either AEA at different concentrations (1 nM, 10 nM, 100 nM) or CAP (10 nM) and the specificity of the reactions was verified by the use of the antagonists SR144528 (10 nM) or CPZ (100 nM), respectively. For each hemolymph pool the positive control was represented by the hemocytes incubated only with Zymosan A. The hemocytes ability to generate ROS was measured by means of luminol-augmented chemiluminescence assay. Each hemolymph pool was adjusted to 10^6^ hemocytes/mL with ice cold ASS. Different aliquots were prepared and then placed in 96-wells microplate. All aliquots were briefly incubated with luminol (Sigma-Aldrich Co., St. Louis, MO, USA) at 1 mM before addition of Zymosan A, AEA (1 nM, 10 nM, 100 nM) and CAP (10 nM). As described above, the antagonists SR144528 (10 nM) or CPZ (100 nM) were used to verify the specificity of the reaction. For each hemolymph pool the positive control was represented by the hemocytes incubated only with luminol and Zymosan A. The luminescence intensity was monitored by luminometer (Packard Fusion/Perkin Elmer, MA, USA) for 1 h. Twenty different hemolymph pools were considered for both phagocytosis assays and three technical replicates were performed for each pool. Data were reported as percentage decrease of the hemocyte ability to spread and to generate ROS, compared to positive control. Data represented the mean values of 20 hemolymph pools.

### 4.9. Statistical Analysis

Data reported in this paper are the means ± S.E.M. of at least three independent experiments, each performed in triplicate. Data were analyzed in the GraphPad Prism statistical PC program (GraphPad Software, La Jolla, CA, USA) using the Mann–Whitney U-test. A level of *p* < 0.05 was considered statistically significant. Apparent dissociation constant (K_d_) and maximum binding (B_max_) values were calculated from saturation curves through nonlinear regression analysis with the Prism 5 program.

## 5. Conclusions

In conclusion, we suggest that ECS is an ancestral signaling system that has been used along evolution as a common chemical language to drive the immune response in the neuroendocrine–immune axis [57,58].

## Figures and Tables

**Table 1:** Endogenous levels and metabolism of AEA, 2-AG, and PEA in the Mediterranean mussel *Mytilus galloprovincialis* hemocytes.

ECS Element	Control	Inhibitor
Endogenous levels of AEA ^a^	1.90 ± 0.16	−
Endogenous levels of 2-AG ^a^	43.5 ± 7.2	−
Endogenous levels of PEA ^a^	0.40 ± 0.07	−
NAPE-PLD ^b^	120.8 ± 0.6	N.A.
FAAH ^b^	66.3 ± 6.6	20.3 ± 3 ^c^
DAGL ^b^	200 ± 3	123 ± 7 ^d^
MAGL ^b^	350 ± 10	252 ± 22 ^e^
NAAA ^b^	96.9 ± 19.6	N.A.

^a^ Expressed as pmol/mg of protein; ^b^ Expressed as pmol/min per mg of protein; ^c^ Inhibited by 0.1 µM URB597; ^d^ Inhibited by 0.1 µM O-3841; ^e^ Inhibited by 20 µM URB602; N.A. Not Available.

## Supplementary Materials

The following are available online at https://www.mdpi.com/article/10.3390/ijms22094954/s1, Table S1: BLAST analysis data.
